# Observations on Ulcers of the Legs and Other Parts, Showing That the Most Obstinate and Intractable Cases May Be Speedily Cured by Mild Methods of Treatment, with Remarks on Scrofulous Disorders

**Published:** 1843-01

**Authors:** 


ART. II.—*Observations on Ulcers of the Legs and other parts, showing that the most obstinate and intractable cases may be speedily cured by mild methods of treatment; to which are appended some remarks on Scrofulous Disorders.* By ARCHIBALD MAXFIELD, Surgeon to the South Hants Infirmary, &c.—*London,* 1842. 8vo, pp. 80.

THE author's assertion is that all ulcers on the legs, which are not strictly constitutional, depend on or are aggravated by disease of the adjacent veins; and that they may all be cured by filling their cavities with sponge or lint, or some mild soft substance, and applying a bandage smoothly and firmly over the whole leg. This is the substance of all that is worth attention in the book.

Had the author had no other object than that of recommending this well-known, though rather neglected, method of treatment, he would have attained his end more certainly by making of his book a short paper, and communicating it to some weekly medical journal. Three pages would have contained all that he really had to say. The appended remarks on scrofula, which contribute twelve pages to the making of the book, seem to be taken from some of the minor elementary works on surgery.

